# Combined Analysis of Endothelial, Hematopoietic, and Mesenchymal Stem Cell Compartments Shows Simultaneous but Independent Effects of Age and Heart Disease

**DOI:** 10.1155/2017/5237634

**Published:** 2017-07-27

**Authors:** Carine Ghem, Lucinara Dadda Dias, Roberto Tofani Sant'Anna, Renato A. K. Kalil, Melissa Markoski, Nance Beyer Nardi

**Affiliations:** ^1^Institute of Cardiology of Rio Grande do Sul, Fundação Universitária de Cardiologia, Av. Princesa Isabel 395, 90040-371 Porto Alegre, RS, Brazil; ^2^Universidade Federal de Ciências da Saúde de Porto Alegre, Rua Sarmento Leite 245, 90050-170 Porto Alegre, RS, Brazil; ^3^Laboratory of Stem Cells and Tissue Engineering, Universidade Luterana do Brasil, Av. Farroupilha 8001, 92425-900 Canoas, RS, Brazil

## Abstract

Clinical trials using stem cell therapy for heart diseases have not reproduced the initial positive results obtained with animal models. This might be explained by a decreased regenerative capacity of stem cells collected from the patients. This work aimed at the simultaneous investigation of endothelial stem/progenitor cells (EPCs), mesenchymal stem/progenitor cells (MSCs), and hematopoietic stem/progenitor cells (HSCs) in sternal bone marrow samples of patients with ischemic or valvular heart disease, using flow cytometry and colony assays. The study included 36 patients referred for coronary artery bypass grafting or valve replacement surgery. A decreased frequency of stem cells was observed in both groups of patients. Left ventricular dysfunction, diabetes, and intermediate risk in EuroSCORE and SYNTAX score were associated with lower EPCs frequency, and the use of aspirin and *β*-blockers correlated with a higher frequency of HSCs and EPCs, respectively. Most importantly, the distribution of frequencies in the three stem cell compartments showed independent patterns. The combined investigation of the three stem cell compartments in patients with cardiovascular diseases showed that they are independently affected by the disease, suggesting the investigation of prognostic factors that may be used to determine when autologous stem cells may be used in cell therapy.

## 1. Introduction

Cardiovascular disease is one of the major causes of death in the world and requires an extended period of treatment, resulting in high medical costs. Since the first experimental application of stem cell therapy in heart diseases [1], a very large number of preclinical animal studies have shown that stem/progenitor cells have the ability to improve cardiac function and reduce infarct size, in ischemic as well as nonischemic cardiomyopathy [2, 3]. Clinical trials were started very soon after that, using mainly bone marrow-derived stem cells (BMSCs). In spite of reports of effectiveness of cell therapy in heart diseases (reviewed in [4]), the translation of preclinical beneficial results into the clinical setting has been limited. As recently summarized in a systematic review of major randomized controlled cell therapy clinical trials for heart diseases [5], most have proven safe but very limited in clinical efficacy. Despite modest improvements of left ventricular ejection fraction, mortality, reinfarction, or rehospitalization rates were not modified [6].

Many considerations have been made about the most adequate types of stem cells to be used for treating cardiovascular diseases [7]. The most obvious difference between preclinical and clinical studies is the source of stem cells. Preclinical studies generally use young, healthy animals in which the condition to be investigated is experimentally induced. Stem cells collected from these animals may represent a completely different population in terms of frequency and therapeutic potential, as compared to autologous cells collected from aged patients with heart failure.

A reduction in the function of stem cells frequencies plays a role in tissue ageing and in diseases [8, 9]. A number of studies have shown that risk factors for cardiovascular disease can affect bone marrow progenitor cells, decreasing the ability of regeneration and reducing the effectiveness of cells derived from patients [10, 11]. Attention has also been given to the role of cardiac stem cells (CSCs). The percentage of c-kit + CSCs was shown to be negatively correlated with age, diabetes mellitus, and coronary heart disease [12], and growth properties of these cells have been suggested as a novel biomarker of the outcome of coronary bypass surgery [13].

Three populations of stem and progenitor cells have particular importance for the treatment of cardiovascular diseases: endothelial progenitor cells (EPCs) in the bone marrow and peripheral blood, mesenchymal stem cells (MSCs) in the bone marrow and all other tissues and organs, and hematopoietic stem cells (HSCs) in the bone marrow. Although the investigations consistently suggest the presence of a dysfunction of these stem cell compartments in clinical situations, many issues have not yet been clarified. First, although the occurrence of this type of dysfunction in EPCs is described, it has not been consistently investigated for other stem cell types. The role of MSCs in heart disease is particularly relevant, in view of the therapeutic potential of this cell type [14]. Second, some relevant clinical situations have not been explored. And finally, no studies have investigated different types of stem cells in the same patients, in order to evaluate the functional capacity or residual numbers of stem cell compartments and their contribution for the disease. This consideration is particularly important when the source of stem cells to be used in cell therapy procedures is defined [15], since autologous stem cells may not be able to regenerate the injured tissues.

To contribute with this important point, the present study conducted a simultaneous investigation of the frequency of three stem cell compartments—endothelial, hematopoietic, and mesenchymal—in the bone marrow of patients with ischemic or valvular heart disease.

## 2. Materials and Methods

### 2.1. Patients

This study included patients referred for coronary artery bypass grafting or valve replacement surgery due to ischemic heart disease (IHD) or nonischemic valvular heart disease (VHD), recruited at Institute of Cardiology of Rio Grande do Sul (RS, Brazil) between May 2011 and June 2012. Exclusion criteria were age lower than 35 years or higher than 70 years, presence of hematologic diseases, cancer, chemotherapy treatment, and previous surgical operation.

Clinical and laboratory data were obtained from medical records, including age, gender, weight (kg), height (m), high blood pressure (defined as use of antihypertensive medication), smoking, diabetes (defined by the use of oral hypoglycemic drugs or insulin), and use of *β*-blocker, aspirin, or statin. Preoperative hematocrit, hemoglobin, and fasting glucose levels were also included. Kidney function was assessed by determining creatinine clearance with the Cockcroft-Gault equation.

The ejection fraction and left ventricular mass were measured by two-dimensional and Doppler echocardiography. Left ventricular (LV) dysfunction was defined as ejection fraction less than 50% [16] and left ventricular hypertrophy as left ventricular mass index >88 g/m^2^ in women and >102 g/m^2^ in men [17]. The mortality risk was estimated by the logistic EuroSCORE [18], and the SYNTAX score was used to score and grade the coronary lesions [19].

### 2.2. Isolation and Cultivation of Bone Marrow Mononuclear Cells

Immediately before sternotomy and with the patient under sedation, approximately 20 mL of bone marrow cells was collected by needle puncture of the sternal manubrium anterior wall. Mononuclear cells (MNCs) were isolated by density centrifugation over Ficoll-Paque Plus (GE Healthcare Life Sciences, Piscataway, NJ) for 40 min at 400*g*, at room temperature. For viability studies, cells were resuspended in RPMI 1640 medium with 100 U/mL penicillin, 0.05 *μ*g/mL streptomycin, and 15% fetal bovine serum (Cultilab, SP, Brazil), and plated onto 96-well plates at 5 × 10^6^ cells/cm^2^. After 1, 3, or 7 days of cultivation, the frequency of viable cells was determined as described above.

All reagents used were from Sigma Chemical Co. (St. Louis, MO), unless otherwise stated. Plasticware was from BD-Brazil (Sao Paulo, Brazil). All cultivations were performed at 37°C in a 5% CO_2_ incubator. Cultures were routinely observed with an inverted phase-contrast microscope (Axiovert 25; Zeiss, Hallbergmoos, Germany). Photomicrographs were taken with a digital camera (AxioCamMRc, Zeiss), using AxioVision 3.1 software (Zeiss).

### 2.3. Analysis of the Endothelial Progenitor Cell Compartment

The EPC compartment in the sternal bone marrow was analyzed by the colony-forming unit (CFU) method [20] and by flow cytometry (below). For the CFU assay, MNCs were resuspended in CFU-Hill medium (StemCell Technologies, Vancouver, Canada), plated on 6-well plates coated with fibronectin (BD-Brazil, SP, Brazil) at a concentration of 5 × 10^5^ cells/cm^2^, and cultured for 48 hours. Nonadherent cells were then collected, resuspended in the same medium, plated in duplicate samples onto fibronectin-coated, 24-wells plates at 5.2 × 10^5^ cells/cm^2^, and cultured for 3 days. After staining with Giemsa, CFU-Hill units, characterized by a central cluster surrounded by elongated cells, were blindly counted and shown as median and interquartile intervals per 10^6^ MNCs.

### 2.4. Analysis of the Hematopoietic Stem Cell Compartment

The HSC compartment was analyzed by a colony assay using methylcellulose-based culture media with cytokines and by flow cytometry (below). For the colony assay, MNCs were resuspended in Methocult H4034 Optimum medium (StemCell Technologies) and plated onto 6-well plates at 2 × 10^4^ cells/cm^2^. After 14 days, colonies were blindly counted as erythroid burst-forming units (BFU-E), colony-forming units-granulocyte/macrophage (CFU-GM), or multipotent myeloid stem cells (CFU-GEMM) and shown as median and interquartile intervals per 10^5^ MNCs.

### 2.5. Analysis of the Mesenchymal Stem Cell Compartment

The MSC compartment was investigated by the colony-forming unit-fibroblast (CFU-F) and by the establishment of conventional MSC cultures, which were then analyzed for immunophenotype and differentiation potential. The CFU-F assay was conducted as previously described [21]. Briefly, MNCs were plated in triplicate samples onto 6-well plates, at 5 × 10^4^ cells/cm^2^, and cultured for 14 days. After staining with May-Grünwald Giemsa, colonies (clusters of ≥30 cells with fibroblastoid morphology) were blindly counted and shown as median and interquartile intervals per 10^6^ MNCs.

To establish MSC cultures, MNCs were resuspended in Dulbecco's modified Eagle's medium (DMEM) with HEPES, 50 U/mL penicillin, 0.05 *μ*g/mL streptomycin, and 15% fetal bovine serum (Cultilab) and plated onto 12-well plates at 2 × 10^6^ cells/cm^2^. Three days later, nonadherent cells were removed. For subculture, the adherent layer was incubated with 0.25% trypsin and 0.01% EDTA and split at ratios empirically determined for two subcultures a week. Cultures were considered successful when reaching the third passage (P3). The plasticity of MSCs was analyzed by incubating P3 or P4 cultures with adipogenic or osteogenic medium as described [22]. Differentiated cells were identified by staining with Oil Red O or Alizarin red, respectively. Cultures were immunophenotyped by flow cytometry (below).

### 2.6. Flow Cytometry

For analysis of the frequency of EPCs and HSCs by flow cytometry in fresh bone marrow samples, 10^5^ MNCs were incubated for 15 min with antibodies specific for CD34 and KDR or CD34 and CD38 (BD-Brazil), respectively. The anti-CD34 antibody was conjugated to phycoerythrin, and the other two antibodies to fluorescein isothiocyanate. After washing for removal of excess antibodies, the cells were analyzed using a FACScalibur cytometer equipped with 488 nm argon laser (Becton Dickinson, San Diego, CA) with the CellQuest software. At least 100,000 events in the lymphocyte gate were collected.

For immunophenotyping of MSCs, P3 or P4 cultures were trypsinized, washed, and incubated for 15 min with specific antibodies for CD34, CD45, CD14, CD90, CD105, CD73, and HLA-DR (BD-Brazil), conjugated to phycoerythrin or fluorescein isothiocyanate. The cells were analyzed in a FACScalibur flow cytometer as above, and 10,000 events were collected.

### 2.7. Statistical Analysis

Statistical analyses were performed with the SPSS software package, version 19.0, and GraphPad Prism 5. Numeric variables are described as mean and standard deviation or median and interquartile range (25–75%). Categorical variables are described as proportions. Categorical variables were analyzed with the Chi-square test, and numeric variables with Student's *t*-test and the Mann–Whitney test. Correlations were analyzed with the Pearson correlation coefficient. A *p* value of <0.05 was considered statistically significant for all comparisons.

## 3. Results

### 3.1. Patient Population

The population studied included 20 patients with IHD and 16 with VHD. As presented in Table 1, mean age was similar in the two groups; there were no significant differences observed in gender or ejection fraction, but the body mass index was lower in IHD patients. Furthermore, a higher use of *β*-blockers, aspirin, and statins was also observed in IHD patients.

Patients were also classified according to the presence or absence of LV dysfunction, with no differences in baseline characteristics (Table 1).

### 3.2. Isolation and Viability of Sternal Bone Marrow Mononuclear Cells

**Figure 1:**
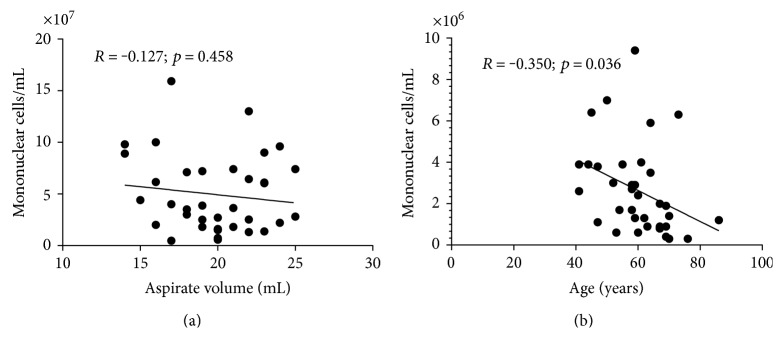
Relationship between the concentration of mononuclear cells and mean volume of the sternal bone marrow collected (a) and age of patients (b).

The mean volume of the sternal bone marrow collected was 20 ± 3 mL, which yielded in average 2.5 ± 1.8 × 10^6^ cells/mL of tissue without significant correlation between MNC concentration and bone marrow aspiration volume (Figure 1(a)). There was however a significant inverse correlation between MNC concentration and age of patients (Figure 1(b)).

The cells were cultured on fibronectin-coated plates, and the viability of adherent and nonadherent cells, evaluated on days 1, 3, and 7, was always higher than 90% (not shown). Some of the samples could not be analyzed by all of the methods employed in this study, mainly for technical reasons.

### 3.3. Endothelial Progenitor Cell Compartment

**Figure 2:**
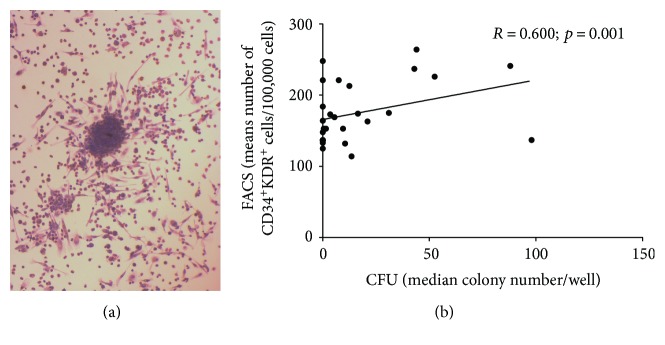
Analysis of the endothelial progenitor cell compartment. (a) CFU-hill colony (original magnification: ×200). (b) Flow cytometry (FACS) and the colony assay (CFU) showed a good correlation in the determination of EPC Frequencies. Scale bar: 100 *μ*m.

CFU-Hill colonies presented the typical morphology of a central cell cluster surrounded by emerging cells (Figure 2(a)), and a good correlation was observed between the colony assay and flow cytometry in determining EPC frequencies (Figure 2(b)). The number of colonies/well showed great variation, but the difference was not statistically significant (Table 2). However, a significantly lower clonogenic potential was observed in samples from patients with LV dysfunction (Table 2). Determination of the frequency of EPCs by flow cytometry (CD34^+^KDR^+^ cells) had a good correlation with the colony assay (Figure 2(b)) and showed similar results in all groups of patients (Table 2).

The cells used in the EPC colony assay were also evaluated for viability, when nonadherent cells were replated on day 2. Similar results were observed in the two groups of samples, with over 99% viability (*p* = 0.957) (not shown).

### 3.4. Hematopoietic Stem Cell Compartment

**Figure 3:**
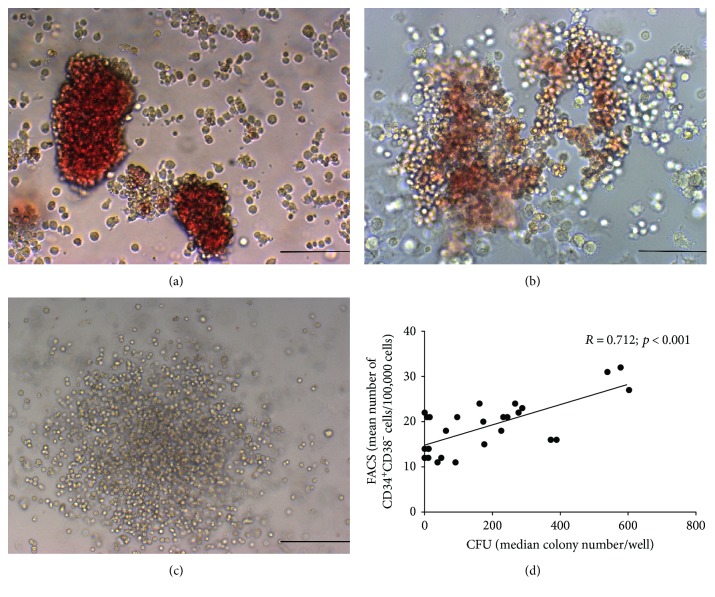
Analysis of the hematopoietic stem cell compartment. (a), (b), and (c) BFU-E, CFU-GEMM, and CFU-GM colonies, respectively, analyzed in the colony assay (original magnification: ×200). (d) Flow cytometry (FACS) and the colony assay (CFU) showed good correlation in the determination of HSC frequencies. Scale bar: 100 *μ*m.

The HSC compartment was analyzed with a colony assay which allowed the identification of different types of precursors in the bone marrow mononuclear fraction (Figures 3(a), 3(b), and 3(c)). As presented in Table 2, IHD samples had in general higher numbers of the three types of colonies, as well as for the total number of colonies, but the differences were not statistically significant. Flow cytometry results for CD34^+^CD38^−^ cell frequency had a good correlation with the colony assay (Figure 3(d)), and although much less variable showed similar results between IHD and VHD patients (Table 2). Results were similar for patients with or without LV dysfunction.

### 3.5. Mesenchymal Stem Cell Compartment

**Figure 4:**
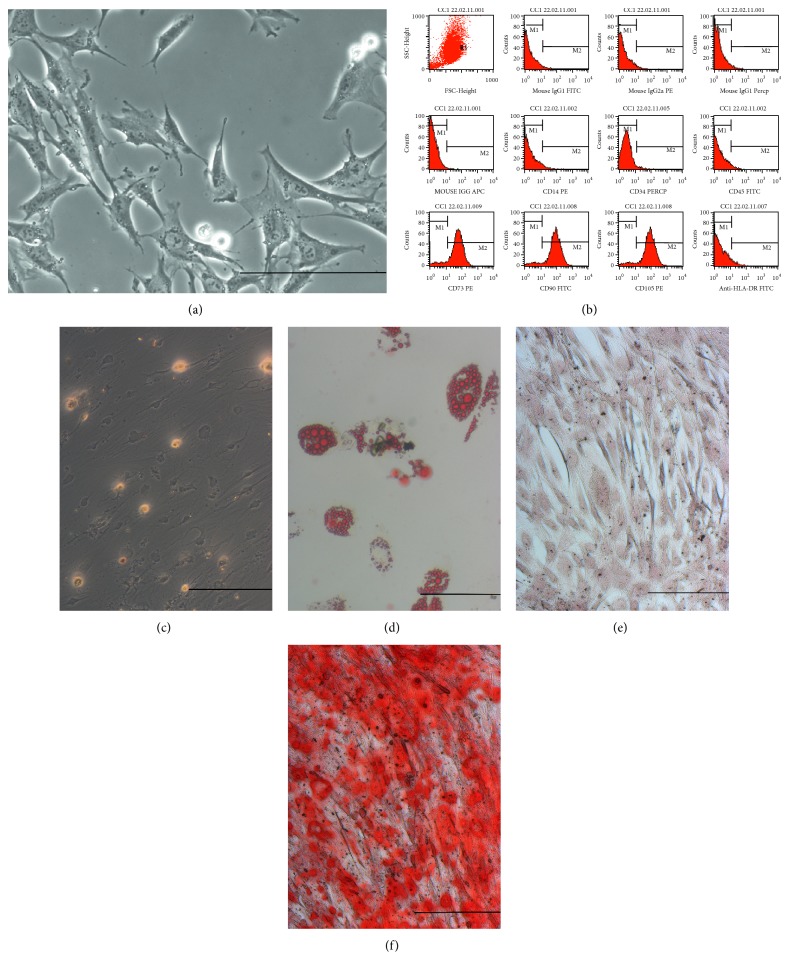
Analysis of the mesenchymal stem cell compartment. (a) Characteristic fibroblastoid morphology of MSC. (b) Immunophenotyping of MSC cultures showing negative results for CD34, CD14, CD45, and HLA-DR, and positive results for CD73, CD90, and CD105. (c), (d) Oil Red O staining of control and adipocyte-differentiated cultures, respectively. (e), (f) Alizarin red staining of control and osteoblast-differentiated cultures, respectively. Original magnification: ×400 (a), ×200 (c)–(f). Scale bar: 100 *μ*m.

The CFU-F assay showed a very low frequency of mesenchymal stem cells in samples from both groups of patients (Table 2). Among the 15 samples from IHD patients, in only one case (7%) the culture was established. For VHD samples, two cases among 14 MSC cultures (14%) were successful. These cultures presented the characteristic fibroblastoid morphology of mesenchymal stem cells (Figure 4(a)). Immunophenotyping of MSC cultures showed low or no expression of hematopoietic markers (CD34, CD14, CD45) and HLA-DR and presence of CD73, CD90, and CD105 (Figure 4(b)). After three weeks in culture with differentiation-inducing media, all MSC cultures differentiated into adipocytes or osteocytes (Figures 4(c), 4(d), and 4(f)).

### 3.6. Distribution of Frequencies of the Stem Cell Compartments

**Figure 5:**
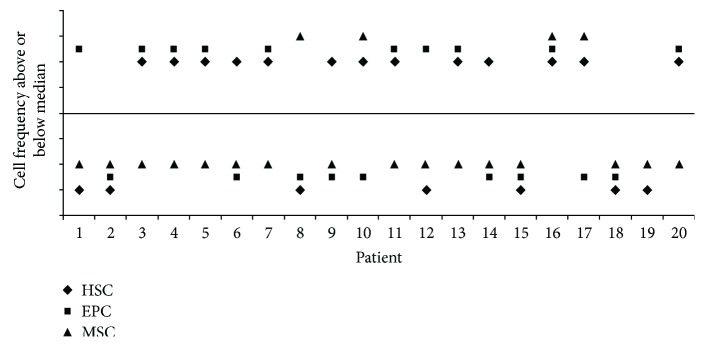
Qualitative individual evaluation of frequencies in the three stem cell compartments. Frequencies were determined by the colony assay in IHD (1 to 12) and VHD (13 to 20) samples. Results of the three colony assays were individually assessed in 20 patients to compare for the occurrence of frequencies higher or lower than the median. In this qualitative analysis, the line represents the median frequency of colony assays, and individual colony frequencies are displayed above or below the corresponding value.

The results of the three colony assays were individually compared in the 20 patients for whom the complete results were available. As shown in Figure 5, in only five cases, the frequency of stem cells from the three compartments is above (*n* = 1) or below (*n* = 4) the median. In all other samples, the distribution of frequencies of stem cells is placed above or below the median line in a variable pattern, considering the characteristics analyzed in the population of patients.

### 3.7. LV Dysfunction and Cardiovascular Risk Factors versus Stem Cell Compartments

**Figure 6:**
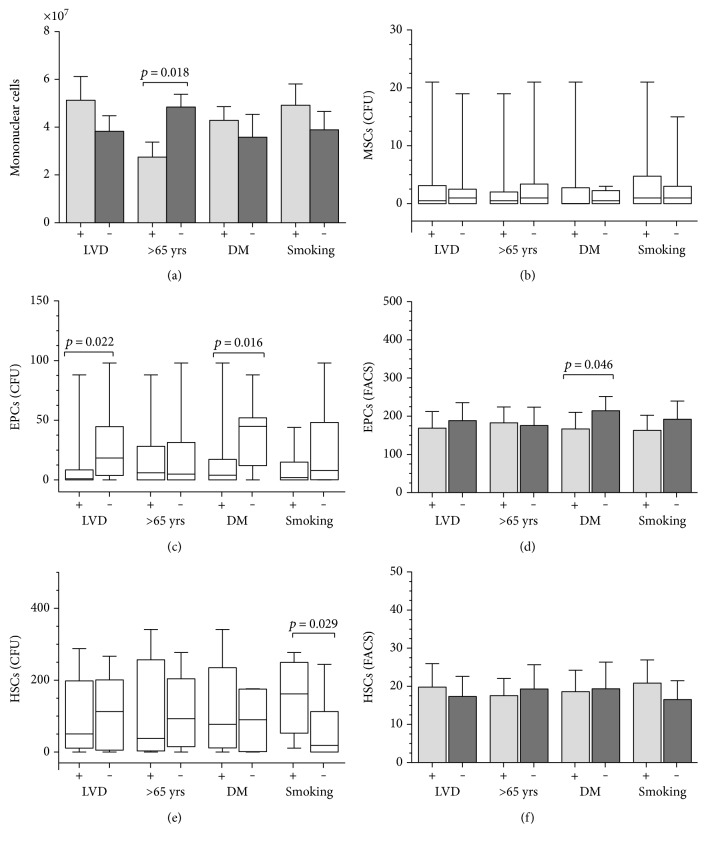
Effect of LV dysfunction (LVD) and cardiovascular risk factors (age over 65 years, diabetes mellitus, and smoking) on bone marrow stem cell compartments. (a) Number of mononuclear cells isolated from the sternal bone marrow. Results of clonogenic assay (CFU) and flow cytometry analyses (FACS) of mesenchymal stem cells (MSCs, (b)), endothelial progenitor cells (EPCs, (c), (d)), and hematopoietic stem cells (HSCs, (e), (f)). Student's *t*-test and Mann–Whitney test. DM: Diabetes mellitus. + or −: presence or absence.

The frequency and function of stem cells from the sternal bone marrow was analyzed according to presence of LV dysfunction, diabetes, and smoking and age greater than 65 years. The number of isolated cells was significantly higher for age below 65 years (Figure 6(a)). Similar frequencies of MSCs were observed in all groups (Figure 6(b)). The clonogenic potential of EPCs was lower in samples from older patients and in the presence of cardiovascular risks, but statistical significance was observed only in the presence of LV dysfunction and diabetes (Figure 6(c)). Their frequency as assessed by flow cytometry was similar for all groups (Figure 6(d)). For HSCs, similar clonogenic (Figure 6(e)) and flow cytometry (Figure 6(f)) results were observed, except for a higher clonogenic potential in samples from smoking patients.

**Figure 7:**
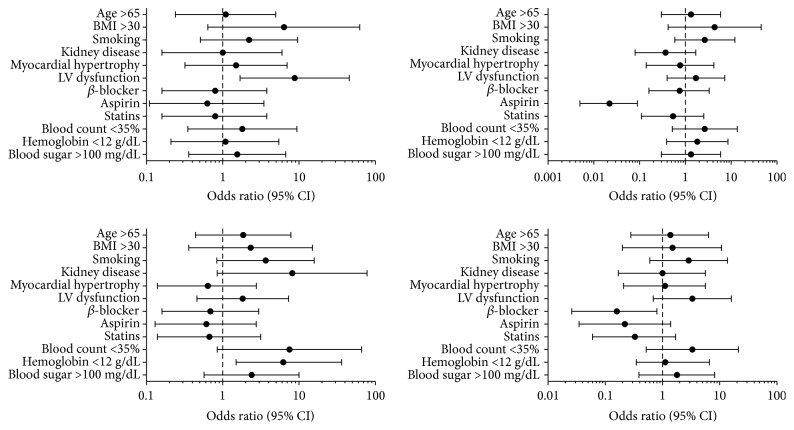
Odds ratio for the frequency and clonogenic potential of compartments of endothelial and hematopoietic stem cells.

A simple logistic regression model was used to identify clinical and laboratory characteristics potentially affecting the frequency and function of EPCs and HSCs in sternal bone marrow samples. The characteristics evaluated were age, body mass index, smoking, renal disease, myocardial hypertrophy, LV dysfunction, use of medications, anemia, and fasting glucose level. The following associations were observed (Figure 7): lower clonogenic potential for EPCs and LV dysfunction (OR = 8.8; 95% CI = 1.69–45.78; *p* = 0.006), increased frequency of CD34^+^KDR^+^ cells and use of aspirin (OR = 0.02; 95% CI = 0.00–0.09; *p* = 0.022), lower clonogenic potential for HSCs and hemoglobin level <12 g/dL (OR = 6.2; 95% CI = 1.5–36.21; *p* = 0.030), and increased frequency of CD34^+^CD38^−^ cells and use of *β*-blockers (OR = 0.16; 95% CI = 0.02–0.80; *p* = 0.043). The frequency and function of MSCs could not be analyzed with this model, due to the low frequency of this cell population in the samples.

An increased frequency of CD34^+^KDR^+^ cells was observed in samples from low-risk patients in both the EuroSCORE and the SYNTAX score, and a higher frequency of CD34^+^CD38^−^ cells was seen in samples from low-risk patients according to the SYNTAX score (Table 3).

## 4. Discussion

This study characterized stem cell compartments in the bone marrow of patients with ischemic or valvular heart disease. The sample included mostly elderly patients with cardiovascular risk factors such as obesity, hypertension, smoking, and diabetes. The groups with ischemic or valvular heart diseases showed similar clinical and laboratory characteristics, differing mainly in features such as the BMI and the use of medications including *β*-blockers, aspirin, and statins used as a preventive measure of cardiovascular events for patients with ischemic heart disease [23].

The MSC compartment showed extremely low cell frequencies in the two types of heart diseases, and successful cultures could be established only from one IHD sample and two VHD samples. These cultures were evaluated for immunophenotype and osteogenic/adipogenic differentiation, showing features typical of MSCs as proposed by the International Society for Cellular Therapy [24]. The methods for isolating and cultivating MSC are very well established and have been used by our group for over 10 years for murine [21, 25], rat [26], canine [27], and human [28, 29] bone marrow- and adipose tissue-derived cells. Therefore, the difficulty in establishing MSC cultures should be explained by intrinsic characteristics of the sample, such as old age and disease.

In the present study, the frequencies of MSCs obtained from sternal bone marrow samples from patients with IHD and VHD were, respectively, 0.6 and 0.2 CFU-F/10^6^ MNCs, significantly lower than those found in younger, normal individuals [30, 31]. Similar results were recently described by Neef et al., who observed a frequency of 5.5 colonies/10^6^ MNCs in a group of patients undergoing elective cardiac surgery, with mean age of 68 years [10]. A decline in the number of CFU-F with the advancement of age has been previously reported, with CFU-F values around four times lower in 21–40-year-old bone marrow donors than in 0–20-year-old donors [32]. MSC cultures established from bone marrow samples from healthy children show high proliferative potential, rapid growth, and better clonogenic potential as compared to bone marrow samples from healthy adults [33]. Similarly, heart disease and other pathological conditions have been shown to decrease MSC frequencies [8, 34]. Animal studies have also shown a decrease in the proliferative potential of MSCs with age [35].

Cell frequencies in the EPC compartments were also similar in the two groups of patients, as shown by flow cytometry and colony assay. A correlation was observed between the number of CFU and the number of CD34^+^KDR^+^ cells assessed by flow cytometry in samples from cardiac patients. In contrast, George et al. [36] did not observe a correlation between the number of EPCs measured by the colony assay and flow cytometry in the peripheral blood of healthy individuals, suggesting that the flow cytometry is more suitable to define the frequency of circulating EPCs, while the number of CFU would reflect their proliferative ability.

In bone marrow samples from healthy donors, the frequency of CD34^+^KDR^+^ cells was reported as 5.43% [37], much higher than the frequencies observed in the present study. We also observed a lower clonogenic potential for the EPC compartment in samples from both groups of patients than that reported for normal bone marrow donors [37].

In our study, the frequency of HSCs was similar in samples from both groups of patients. The frequency of CD34^+^CD38^−^ cells observed in IHD and VHD samples was lower than the frequency reported in bone marrow samples from healthy individuals [38], showing that the hematopoietic stem cell compartment is also affected in heart disease patients. The colony assay also showed low numbers of HSCs in the present study, as compared to values in healthy younger individuals [39]. In a comparison of samples from heart disease patients and healthy controls, lower numbers of CFU-GM were observed in the first group [40]. Function, besides frequency, may be affected by age and disease. The migratory response and clonogenic potential of bone marrow-derived circulating progenitor cells were shown to be impaired in patients with cardiovascular disease, suggesting that the mobilization and homing of stem cells may be also affected by heart disease [41].

The characteristics of these three cell populations in the iliac crest bone marrow are well known, but few studies have compared cells isolated from different sites, particularly the sternum. MSCs isolated from the equine sternum and ilium showed similar characteristics [42, 43], but a significantly faster proliferation rate has been reported for sternal than for ilial cells [44]. In sheep, the sternum was considered as an equally good source of bone marrow MSCs, with cell division cycle and proliferative potential similar to the cells from the iliac bones [45]. Human MSCs isolated from the iliac crest, sternum, and vertebrae bone marrow have also shown similar immunophenotype but different growth/differentiation potentials and homeobox gene expression, suggesting that they do not represent equivalent cell sources for therapeutic applications [46]. Gradual loss of ability to proliferate and a morphological conversion of senescence tendency have also been reported for human sternal MSCs [47].

EPCs have been mainly analyzed as circulating cells, due to the association between this variable and cardiovascular risk [48], and are more frequent in the bone marrow than in nonmobilized cytapheresis peripheral blood [49]. For HSCs, initial studies showed that the concentration of CFUs in murine sternal marrow is about 40% less than in the marrow of lumbar vertebrae and femora [50]. The comparison cord blood, bone marrow, and peripheral blood have shown significant differences in mean cell density values [51], as well as gene and miRNA expression profiles [52, 53], suggesting diversity in biological processes such as cell cycle regulation and cell motility.

Many of the risk factors for cardiovascular disease are well established, and their effect on stem cell compartments has been shown [12, 13]. The present results, showing that the number of mononuclear cells isolated from the bone marrow is lower in patients older than 65 years, support previous studies on bone marrow ageing [11] and its relationship with a decrease in the frequency of MSCs [32], HSCs [54], and EPCs [48]. A relationship was also observed between diabetes and lower frequency and clonogenic potential of EPCs, as already described [55], showing the prejudicial impact of this pathology on the endothelial precursor cell compartment. An interesting result was the higher clonogenic potential of HSCs in samples from smoking patients, since smoking is a well-established risk factor for cardiovascular disease. Similar results have been previously reported in an animal study [56], which however showed no increase in cellular function.

The SYNTAX score was developed to quantify the complexity of coronary lesions and is used to guide treatment of patients with ischemic heart disease [57]. The score can also be used to predict long-term major cardiovascular events after revascularization [58]. In our study, patients with a higher SYNTAX score, which is an indicative of higher atherosclerotic burden and more severe ischemic heart disease, had a lower number of CD34^+^KDR^+^ and CD34^+^CD38^−^ cells.

The present study has some limitations, such as a relatively small sample and the lack of a control group matched for age. The problem of a control group is very difficult to solve in this kind of study: the bone marrow from healthy donors is not adequate, and ethical reasons do not allow sternal bone marrow collection from aged cardiac patients or healthy volunteers. Nevertheless, our results support previous reports showing decreased frequencies of stem cells in aged, diseased individuals [59] and presents in a more comprehensive manner the concept of how age and disease may affect stem cell compartments. To our knowledge, this is the first time that different types of stem cell compartments are analyzed in patients with cardiovascular diseases, which has made it possible to observe that they are simultaneously, but independently affected by age and disease. Although all three types of stem cells presented lower frequencies than those reported in the literature for young, normal individuals, a large variation was observed among them, and the compartments distributed above or below the median frequency in an independent manner. This suggests that the MSC, HSC, and EPC compartments are independently affected in aged patients with heart disease.

The use of allogeneic mesenchymal stem cells has already been proven safe in patients with myocardial infarction [60, 61]. Our results added to other studies showing quantitative and functional limitations of stem cell compartments in aged patients with heart disease. Further clinical studies using allogeneic, cultured mesenchymal stem cells from healthy donors, feasible due to their immunoregulatory properties [62], should establish their therapeutic potential in heart failure.

## Figures and Tables

**Table 1:** Baseline characteristics of the study population.

Characteristics	Heart disease			LV dysfunction		
	IHD (*n* = 20)	VHD (*n* = 16)	*p* ^∗^	Presence (*n* = 17)	Absence (*n* = 19)	*p* ^∗^
Age (years)	61.0 ± 8.5	63.0 ± 8.9	0.977	63 ± 8.7	59 ± 9.4	0.382
Male (*n*)	15	9	0.236	14	11	0.160
BMI (kg/m^2^)	26.2 ± 2.8	27.6 ± 8.92	0.015	27 ± 6.3	25 ± 3.1	0.526
Hypertension (%)	94.4	92.9	0.854	88	80	0.357
Smoking (%)	66.7	80	0.222	55	45	0.335
Diabetes (%)	80	86.7	0.605	83	75	0.504
LV EF (%)	55.6 ± 13.7	56.2 ± 13.3	0.749	43 ± 4.3	67 ± 6.5	<0.001
LV mass	110 ± 30.16	131 ± 46.39	0.422	140 ± 26.1	104 ± 22.2	0.045
Hypertrophy (*n*)	11	12	0.301	13	8	0.673
*β*-blockers (%)	90	31.3	<0.001	76	63	0.401
Aspirin (%)	90	37.5	<0.001	72	63	0.542
Statins (%)	85	43.8	0.009	82	73	0.615
Hematocrit (%)	38.6 ± 6.6	38.8 ± 4.6	0.910	38 ± 5.8	39.2 ± 5.7	0.693
Hemoglobin (g/dL)	12.7 ± 2.1	12.8 ± 1.6	0.904	12.4 ± 1.9	12.8 ± 1.9	0.793
Leukocytes (×10^3^/*μ*L)	9.6 ± 3.9	13.0 ± 2.3	0.106	7.9 ± 3.1	6.7 ± 2.5	0.544
Glucose (mg/dL)	133.6 ± 36.4	141.8 ± 63.7	0.629	129.8 ± 49.8	138.7 ± 50.1	0.303
Creatinine (mg/dL)	1.0 ± 0.4	1.0 ± 0.3	0.772	1.02 ± 0.4	1.07 ± 0.3	0.665
Sodium (mEq/L)	137.9 ± 3.6	138.8 ± 2.6	0.392	138.5 ± 3.38	138.1 ± 2.99	0.687
Potassium (mEq/L)	4.4 ± 0.5	4.3 ± 0.4	0.600	4.42 ± 0.4	4.31 ± 0.5	0.477

BMI: body mass index; EV: ejection fraction; IHD: ischemic heart disease; LV: left ventricular; VHD: vascular heart disease. The results are presented as mean and standard deviation or median and interquartile range. ^∗^*p* value, Student's *t*-test and chi-square test.

**Table 2:** Frequency of endothelial progenitor cells, hematopoietic stem cells, and mesenchymal stem cells in the sternal bone marrow samples from patients with ischemic or valvular heart disease, in presence or absence of left ventricular dysfunction. Cells were analyzed by colony assay (CFU) and flow cytometry (FACS).

Cell population (assay)	Heart disease			LV dysfunction		
	IHD (*n* = 20)	VHD (*n* = 16)	*p* ^∗^	Presence (*n* = 17)	Absence (*n* = 19)	*p* ^∗^
EPCs (CFU)	1.9 (0.0–6.5)	0.6 (0.0–7.5)	0.675	1 (0–11)	18 (4–44)	0.038
EPCs (FACS)	174.1 ± 40.2	182.7 ± 51.7	0.611	171 ± 43.4	187 ± 45.6	0.341
HSCs (CFU)						
BFU-E	6.5 (1–23)	1 (0–19)	0.411	21 (1–44)	9 (0–48)	0.727
CFU-GM	22 (6–46)	15.5 (2–46)	0.710	45 (9–95)	44 (7–94)	0.960
CFU-GEMM	54 (14–86)	18 (1–91)	0.313	67 (5–172)	76 (7–266)	0.881
HSCs (CFU)—total colonies	86 (22–154)	48 (5–144)	0.520	93 (14–309)	167 (13–322)	0.855
HSCs (FACS)	19.7 ± 6.6	17.6 ± 4.6	0.347	19.5 ± 6.3	17.9 ± 5.4	0.442
MSCs (CFU)	0.6 (0.3–0.9)	0.2 (0–0.7)	0.400	1 (1–1.5)	3 (1–6)	0.308

BFU-E: erythroid burst-forming units; CFU-GM: colony-forming units-granulocyte/macrophage: CFU-GEMM: multipotent myeloid stem cells; EPCs: endothelial progenitor cells; EV: ejection fraction; HSCs: hematopoietic stem cells; IHD: ischemic heart disease; LV: left ventricular; MSCs: mesenchymal stem cells; VHD: vascular heart disease. The results are presented as mean and standard deviation, or median and interquartile range. ^∗^*p* value, Student's *t*-test and chi-square or Mann–Whitney test.

**Table 3:** Frequency of endothelial progenitor cells, hematopoietic stem cells, and mesenchymal stem cells in the sternal bone marrow samples from patients classified according to the EuroSCORE and SYNTAX score. Cells were analyzed by colony assay (CFU) and flow cytometry (FACS).

Cell population (assay)	EuroSCORE			SYNTAX score		
	Low risk (*n* = 28)	Intermediate risk (*n* = 8)	*p* ^∗^	Low risk (*n* = 13)	Intermediate risk (*n* = 10)	*p* ^∗^
EPCs (CFU)	1.40 (0–5.0)	0.3 (0–14.0)	0.826	1.5 (0–15.5)	1.0 (0–8.6)	0.980
EPCs (FACS)	190.3 ± 62.4	174.0 ± 39.0	0.012	186.0 ± 49.0	158.0 ± 29.0	0.021
HSCs (CFU)	81.0 (8.0–230.0)	68.5 (5.0–142.5)	0.862	81.0 (9.5–220.0)	52.0 (6.0–127.5)	0.685
HSCs (FACS)	20.3 ± 4.5	18.0 ± 6.0	0.083	21.0 ± 9.0	17.0 ± 4.0	0.005
MSCs (CFU)	0.6 (0–6.0)	0.2 (0–0.4)	0.200	0.8 (0–10)	0.4 (0–4.0)	0.824

EPCs: endothelial progenitor cells; HSCs: hematopoietic stem cells; MSCs: mesenchymal stem cells. The results are presented as mean and standard deviation, or median and interquartile range. ^∗^*p* value, Student's *t*-test and Mann–Whitney test.

## References

[1] Orlic D., Kajstura J., Chimenti S. (2001). Bone marrow cells regenerate infarcted myocardium. *Nature*.

[2] Sanganalmath S. K., Bolli R. (2013). Cell therapy for heart failure: a comprehensive overview of experimental and clinical studies, current challenges, and future directions. *Circulation Research*.

[3] Kim J., Shapiro L., Flynn A. (2015). The clinical application of mesenchymal stem cells and cardiac stem cells as a therapy for cardiovascular disease. *Pharmacology & Therapeutics*.

[4] Fisher S. A., Doree C., Mathur A., Taggart D. P., Martin-Rendon E. (2016). Stem cell therapy for chronic ischaemic heart disease and congestive heart failure. *Cochrane Database of Systematic Reviews*.

[5] Nguyen P. K., Rhee J. W., Wu J. C. (2016). Adult stem cell therapy and heart failure, 2000 to 2016: a systematic review. *JAMA Cardiology*.

[6] Pfister O., Della Verde G., Liao R., Kuster G. M. (2014). Regenerative therapy for cardiovascular disease. *Translational Research*.

[7] Musa S., Xin L. Z., Govindasamy V., Fuen F. W., Kasim N. H. (2014). Global search for right cell type as a treatment modality for cardiovascular disease. *Expert Opinion on Biological Therapy*.

[8] Li Y., Charif N., Mainard D., Bensoussan D., Stoltz J. F., de Isla N. (2014). Donor’s age dependent proliferation decrease of human bone marrow mesenchymal stem cells is linked to diminished clonogenicity. *Bio-Medical Materials and Engineering*.

[9] Rurali E., Bassetti B., Perrucci G. L. (2016). BM ageing: implication for cell therapy with EPCs. *Mechanisms of Ageing and Development*.

[10] Neef K., Choi Y. H., Weichel A. (2012). The influence of cardiovascular risk factors on bone marrow mesenchymal stromal cell fitness. *Cytotherapy*.

[11] Mendelsohn A. R., Larrick J. W. (2013). Rejuvenation of adult stem cells: is age-associated dysfunction epigenetic?. *Rejuvenation Research*.

[12] Hu S., Yan G., He W., Liu Z., Xu H., Ma G. (2014). The influence of disease and age on human cardiac stem cells. *Annals of Clinical Biochemistry*.

[13] D'Amario D., Leone A. M., Iaconelli A. (2014). Growth properties of cardiac stem cells are a novel biomarker of patients’ outcome after coronary bypass surgery. *Circulation*.

[14] Meirelles L. S., Nardi N. B. (2009). Methodology, biology and clinical applications of mesenchymal stem cells. *Frontiers in bioscience (Landmark edition)*.

[15] Deutsch M. A., Sturzu A., Wu S. M. (2013). At a crossroad: cell therapy for cardiac repair. *Circulation Research*.

[16] McMurray J. J., Adamopoulos S., Anker S. D. (2012). ESC guidelines for the diagnosis and treatment of acute and chronic heart failure 2012: the task force for the diagnosis and treatment of acute and chronic heart failure 2012 of the European Society of Cardiology. Developed in collaboration with the heart failure association (HFA) of the ESC. *European Journal of Heart Failure*.

[17] Lang R. M., Bierig M., Devereux R. B. (2005). Recommendations for chamber quantification: a report from the American Society of Echocardiography’s guidelines and standards committee and the chamber quantification writing group, developed in conjunction with the European Association of Echocardiography, a branch of the European Society of Cardiology. *Journal of the American Society of Echocardiography*.

[18] Nashef S. A., Roques F., Michel P., Gauducheau E., Lemeshow S., Salamon R. (1999). European system for cardiac operative risk evaluation (EuroSCORE). *European Journal of Cardio-Thoracic Surgery*.

[19] Serruys P. W., Onuma Y., Garg S. (2009). Assessment of the SYNTAX score in the Syntax study. *EuroIntervention*.

[20] Hill J. M., Zalos G., Halcox J. P. (2003). Circulating endothelial progenitor cells, vascular function, and cardiovascular risk. *The New England Journal of Medicine*.

[21] da Silva M. L., Chagastelles P. C., Nardi N. B. (2006). Mesenchymal stem cells reside in virtually all post-natal organs and tissues. *Journal of Cell Science*.

[22] Diesel L. F., dos Santos B. P., Bellagamba B. C. (2015). Stability of reference genes during tri-lineage differentiation of human adipose-derived stromal cells. *Journal of Stem Cells*.

[23] Smith S. C., Benjamin E. J., Bonow R. O. (2011). AHA/ACCF secondary prevention and risk reduction therapy for patients with coronary and other atherosclerotic vascular disease: 2011 update: a guideline from the American Heart Association and American College of Cardiology Foundation endorsed by the World Heart Federation and the Preventive Cardiovascular Nurses Association. *Journal of the American College of Cardiology*.

[24] Dominici M., Le Blanc K., Mueller I. (2006). Minimal criteria for defining multipotent mesenchymal stromal cells. The International Society for Cellular Therapy position statement. *Cytotherapy*.

[25] Meirelles L. S., Nardi N. B. (2003). Murine marrow-derived mesenchymal stem cell: isolation, in vitro expansion, and characterization. *British Journal of Haematology*.

[26] de Macedo Braga L. M., Lacchini S., Schaan B. D. (2008). In situ delivery of bone marrow cells and mesenchymal stem cells improves cardiovascular function in hypertensive rats submitted to myocardial infarction. *Journal of Biomedical Science*.

[27] Marx C., Silveira M. D., Selbach I. (2014). Acupoint injection of autologous stromal vascular fraction and allogeneic adipose-derived stem cells to treat hip dysplasia in dogs. *Stem Cells International*.

[28] Ahmadbeigi N., Soleimani M., Babaeijandaghi F. (2012). The aggregate nature of human mesenchymal stromal cells in native bone marrow. *Cytotherapy*.

[29] Markarian C. F., Frey G. Z., Silveira M. D. (2014). Isolation of adipose-derived stem cells: a comparison among different methods. *Biotechnology Letters*.

[30] Bocelli-Tyndall C., Bracci L., Spagnoli G. (2007). Bone marrow mesenchymal stromal cells (BM-MSCs) from healthy donors and auto-immune disease patients reduce the proliferation of autologous- and allogeneic-stimulated lymphocytes in vitro. *Rheumatology (Oxford, England)*.

[31] Castro-Malaspina H., Gay R. E., Resnick G. (1980). Characterization of human bone marrow fibroblast colony-forming cells (CFU-F) and their progeny. *Blood*.

[32] Stolzing A., Jones E., McGonagle D., Scutt A. (2008). Age-related changes in human bone marrow-derived mesenchymal stem cells: consequences for cell therapies. *Mechanisms of Ageing and Development*.

[33] Choumerianou D. M., Martimianaki G., Stiakaki E., Kalmanti L., Kalmanti M., Dimitriou H. (2010). Comparative study of stemness characteristics of mesenchymal cells from bone marrow of children and adults. *Cytotherapy*.

[34] Kornicka K., Marycz K., Tomaszewski K. A., Marędziak M., Śmieszek A. (2015). The effect of age on osteogenic and adipogenic differentiation potential of human adipose derived stromal stem cells (hASCs) and the impact of stress factors in the course of the differentiation process. *Oxidative Medicine and Cellular Longevity*.

[35] Kretlow J. D., Jin Y. Q., Liu W. (2008). Donor age and cell passage affects differentiation potential of murine bone marrow-derived stem cells. *BMC Cell Biology*.

[36] George J., Shmilovich H., Deutsch V., Miller H., Keren G., Roth A. (2006). Comparative analysis of methods for assessment of circulating endothelial progenitor cells. *Tissue Engineering*.

[37] Tura O., Barclay G. R., Roddie H., Davies J., Turner M. L. (2007). Absence of a relationship between immunophenotypic and colony enumeration analysis of endothelial progenitor cells in clinical haematopoietic cell sources. *Journal of Translational Medicine*.

[38] Hao Q. L., Shah A. J., Thiemann F. T., Smogorzewska E. M., Crooks G. M. (1995). A functional comparison of CD34+CD38- cells in cord blood and bone marrow. *Blood*.

[39] Lanza F., Campioni D., Punturieri M. (2003). In vitro assessment of bone marrow endothelial colonies (CFU-En) in non-Hodgkin’s lymphoma patients undergoing peripheral blood stem cell transplantation. *Bone Marrow Transplantation*.

[40] Heeschen C., Lehmann R., Honold J. (2004). Profoundly reduced neovascularization capacity of bone marrow mononuclear cells derived from patients with chronic ischemic heart disease. *Circulation*.

[41] Bozdag-Turan I., Turan R. G., Paranskaya L. (2012). Correlation between the functional impairment of bone marrow-derived circulating progenitor cells and the extend of coronary artery disease. *Journal of Translational Medicine*.

[42] Adams M. K., Goodrich L. R., Rao S. (2013). Equine bone marrow-derived mesenchymal stromal cells (BMDMSCs) from the ilium and sternum: are there differences?. *Equine Veterinary Journal*.

[43] Kisiday J. D., Goodrich L. R., McIlwraith C. W., Frisbie D. D. (2013). Effects of equine bone marrow aspirate volume on isolation, proliferation, and differentiation potential of mesenchymal stem cells. *American Journal of Veterinary Research*.

[44] Delling U., Lindner K., Ribitsch I., Jülke H., Brehm W. (2012). Comparison of bone marrow aspiration at the sternum and the tuber coxae in middle-aged horses. *Canadian Journal of Veterinary Research*.

[45] Harker G. J., Zbroja R. A., Wass J., Vincent P. C., Stephens F. O. (1983). Cell cycle homogeneity in bone marrow samples from different sites: flow cytometric evaluation of multiple samples from sheep. *Experimental Hematology*.

[46] Picchi J., Trombi L., Spugnesi L. (2013). HOX and TALE signatures specify human stromal stem cell populations from different sources. *Journal of Cellular Physiology*.

[47] Hsu C. P., Wang J. S., Hung S. C., Lai S. T., Weng Z. C., Chiu R. C. J. (2010). Human sternal mesenchymal stem cells: isolation, characterization and cardiomyogenic differentiation. *Acta Cardiologica Sinica*.

[48] Koller L., Hohensinner P., Sulzgruber P. (2016). Prognostic relevance of circulating endothelial progenitor cells in patients with chronic heart failure. *Thrombosis and Haemostasis*.

[49] Tournois C., Pignon B., Sevestre M. A. (2017). Cell therapy in critical limb ischemia: a comprehensive analysis of two cell therapy products. *Cytotherapy*.

[50] Schoeters G. E., Vanderboroght O. L. (1980). Haemopoietic stem cell concentration and CFUs in DNA synthesis in bone marrow from different bone regions. *Experientia*.

[51] Araújo A. B., Angeli M. H., Salton G. D., Furlan J. M., Schmalfuss T., Röhsig L. M. (2017). Absolute density of different sources of hematopoietic progenitor cells: bone marrow, peripheral blood stem cell and umbilical cord blood. *Cytotherapy*.

[52] Wang T. Y., Chang S. J., Chang M. D., Wang H. W. (2009). Unique biological properties and application potentials of CD34+ CD38- stem cells from various sources. *Taiwanese Journal of Obstetrics & Gynecology*.

[53] Báez A., Martín-Antonio B., Piruat J. I. (2014). Gene and miRNA expression profiles of hematopoietic progenitor cells vary depending on their origin. *Biology of Blood and Marrow Transplantation*.

[54] Cohen K. S., Cheng S., Larson M. G. (2013). Circulating CD34(+) progenitor cell frequency is associated with clinical and genetic factors. *Blood*.

[55] Tepper O. M., Galiano R. D., Capla J. M. (2002). Human endothelial progenitor cells from type II diabetics exhibit impaired proliferation, adhesion, and incorporation into vascular structures. *Circulation*.

[56] Chang E., Forsberg E. C., Wu J. (2010). Cholinergic activation of hematopoietic stem cells: role in tobacco-related disease?. *Vascular Medicine*.

[57] Windecker S., Kolh P., Alfonso F. (2014). 2014 ESC/EACTS guidelines on myocardial revascularization: the task force on myocardial revascularization of the European Society of Cardiology (ESC) and the European Association for Cardio-Thoracic Surgery (EACTS) developed with the special contribution of the European Association of Percutaneous Cardiovascular Interventions (EAPCI). *European Heart Journal*.

[58] Mohr F. W., Morice M. C., Kappetein A. P. (2013). Coronary artery bypass graft surgery vs. percutaneous coronary intervention in patients with three-vessel disease and left main coronary disease: 5-year follow-up of the randomised, clinical SYNTAX trial. *Lancet*.

[59] Goichberg P., Kannappan R., Cimini M. (2013). Age-associated defects in EphA2 signaling impair the migration of human cardiac progenitor cells. *Circulation*.

[60] Hare J. M., Traverse J. H., Henry T. D. (2009). A randomized, double-blind, placebo-controlled, dose-escalation study of intravenous adult human mesenchymal stem cells (prochymal) after acute myocardial infarction. *Journal of the American College of Cardiology*.

[61] Suncion V. Y., Ghersin E., Fishman J. E. (2014). Does transendocardial injection of mesenchymal stem cells improve myocardial function locally or globally?: an analysis from the percutaneous stem cell injection delivery effects on neomyogenesis (POSEIDON) randomized trial. *Circulation Research*.

[62] Le Blanc K., Tammik C., Rosendahl K., Zetterberg E., Ringdén O. (2003). HLA expression and immunologic properties of differentiated and undifferentiated mesenchymal stem cells. *Experimental Hematology*.

